# Potentiality Assessment of the Acetylcholinesterase-Inhibitory Activity of Olive Oil with an Additive Edible Insect Powder

**DOI:** 10.3390/molecules28145535

**Published:** 2023-07-20

**Authors:** Joanna Grzelczyk, Ilona Gałązka-Czarnecka, Joanna Oracz

**Affiliations:** Institute of Food Technology and Analysis, Faculty of Biotechnology and Food Sciences, Lodz University of Technology, 90-537 Lodz, Poland; ilona.galazka-czarnecka@p.lodz.pl

**Keywords:** Alzheimer’s disease, edible insects, olive oil, bioactive compounds, acetylcholinesterase

## Abstract

Edible insects (*Alphitobius diaperinus* Panzer, *Gryllus campestris*, *Tenebrio molitor*, *Chorthippus biguttulus*) are rich in nutrients that potentially inhibit acetylcholinesterase (AChE), but also improve cognition. The aim of this study was to evaluate four varied species of freeze-dried edible insects (purchased from a store); their nutrient composition, including fat, total phenolic compounds, vitamins, and antioxidant properties; and the potential inhibitory effect of AChE. An additional goal was to obtain olive oil with the addition of edible insects. Such oil was characterized by high oxidizing properties and showed high affinity to AChE. The results showed that mealworms and grasshoppers had the highest content of fats (PUFA/SFA) and phenolic compounds. These insects also showed a high content of vitamins, which correlated with the highest affinity for AChE. Therefore, they were added as a functional additive to olive oil. Olive oil with the addition of edible insects showed a higher affinity for AChE and enriched the olive oil with vitamin C and B vitamins.

## 1. Introduction

The main cause of Alzheimer’s disease (AD) is described by the cholinergic hypothesis as a significant deficiency of acetyltransferase in the hippocampus and neocortex [1]. Acetylcholinesterase (AChE) catalyzes the hydrolysis reaction of Ach [1,2,3,4]. AChE is responsible for the direct hydrolysis of choline esters through the covalent bonding of hydrogen from serine to histidine. Excessive hydrolysis of choline esters contributes to lowering the concentration of acetylcholine (ACh) below normal levels, leads to the accumulation of β-amyloid, and influences the formation of fibrosis, leading to inflammation [3,5].

A person with AD may find drinking or eating food difficult as the disease progresses, which leads to malnutrition, a lack of strength in performing daily activities and consequently to death [2,3,4,5]. The inhibition of acetylcholine breakdown by AChE will delay the development of the disease through improving memory and reducing beta-amyloid and phosphorylated tau deposits in the brain [3,4,5]. That is why it is important to find a food source that is easy to eat, with high nutritional value, and additionally with bioactive ingredients that will reduce the activity of AChE enzymes.

Many scientific reports indicate that regular consumption of olive oil can prevent the pathogenesis of AD. Olive oil regulates the vascular risk of the disease, thanks to which we slow down the development of the disease through improving cognitive functions. Olive oil is a major source of fatty acids and phenolic fractions [6]. The main compound contributing to reducing the risk of dementia is a synergic effect of omega-3 fatty acids and polyphenols. Therefore, in these studies, it was decided to test the oil with the addition of insects that will enrich the nutritional properties of olive oil [7].

Edible insects are perceived by many people as a contradictory food. We might meet people with such a conviction especially in countries where religion or culture does not allow their consumption [8,9]. However, people in the world are more and more open to new flavors and often decide to try new products and decide to break cultural barriers [8,9]. The prevalence of edible insects in European countries occurred mainly when EFSA (No 2015/2283) recognized insects as a safe food. EFSA includes, among others, *Tenebrio molitor* [10,11]. In the United Kingdom, France, the Netherlands, and Belgium, the sale of edible insects has been carried out for several years. On the other hand, people who are sensitive to the appearance of food may reject the appearance of insects. For such people, it is good to try a dish that contains the addition of insects in ground form, e.g., pasta made from flour from worms or baked goods [12].

Many people ask themselves whether insects are a safe food or do not transmit diseases. When choosing edible insects from known sources, e.g., EFSA, in addition to insects from stores, the consumer must have additional tests required by state institutions carried out. During the production of freeze-dried insects, flours and other products made from insects undergo microbiological tests, but the hazard analysis and critical control points (HACCP) system must also be implemented [13].

Edible insects are all a rich source of wholesome protein. Depending on the species, dried insects can contain up to 60% protein. They are also a great source of iron—per 100 g, they provide more of this mineral than chicken or pork [14]. Depending on the species, insects contain different amounts of essential amino acids for the human body. Through maintaining a balanced diet, we can provide an appropriate amount of amino acids from insects. For example, the content of arginine is higher in the insect *Blatta lateralis* than in meat (pork or beef) [15].

The nutritional value of edible insects is diverse mainly because of the farming conditions, variability of species, origin of the insect, diet, and stage of metamorphosis [10,16,17].

The aim of this study was to evaluate four varied species of freeze-dried edible insects (purchased from store); their nutrient composition, including fat, total phenolic compounds, vitamins, and antioxidant properties; and the potential inhibitory effect of AChE. In addition, the possibility of obtaining olive oil with high antioxidant and AChE-inhibiting properties through the addition of insects was investigated.

## 2. Results and Discussion

### 2.1. Fatty Acid Composition and Lipid Nutritional Indices of an Edible Insect

The conducted research shows that edible insects are characterized by diversity in the fatty acids present (Table 1).

The highest number of compounds was found for house crickets (28 compounds) and four less for mealworms and grasshoppers. On the other hand, the lowest number of detected compounds was found in buffalo worms. Buffalo worms did not contain fatty acids such as behenic acid (C22:0), lignoceric acid (C24:00), erucic acid (C22:1), eicosadienonic acid (C20:2), eicosatrienonic acid (C20:3), arachidonic acid (C20:4), eicosapentaenoic acid (C20:5), and docosahexaenoic acid (C22:6). House crickets did not include tricosanoic acid (C23:0), C20:4, or C22:5; mealworms did not contain heneicosanoic acid (C21:0), C23:0, C24:0, or C22:1; grasshoppers did not contain C24:0, C22:1, C20:5, or C22:6.

Among the saturated fatty acids in insect lipids, omega-9 fatty acid (C 18:1) accounted for the largest share. Mealworms (37.36 g/100 g d.m.) have the highest C18:1 content, followed by buffalo worms (33.13 g/100 g d.m.), then grasshoppers (30.00 g/100 g d.m.). On the other hand, domestic crickets showed about 10 times lower content of the abovementioned acid. An interesting fact is that the highest amount of omega-6 acids (C18:2) were recorded in mealworms (30.22 g/100 g d.m.), compared to 5–6 times less in grasshoppers and buffalo worms, while as much as 29 times less in house crickets (Table 1).

On the other hand, buffalo worms and mealworms were characterized by comparable content of C16:0 of about 5.45–5.85 g/100 g d.m., whereas house crickets contained 4.84 g/100 g d.m. (Table 1), while grasshoppers were characterized by the lowest content of palmitic acid (3.46 g/100 g d.m). According to Udomsil et al. [18], the palmitic acid content in domestic crickets is in the range 5.87–9.26 g/100 g d.m., depending on the species. This suggests that the use of freeze-drying processes lowers the C16:0 content [18]. The results obtained show a low palmitic acid content in the examined insects. The palmitic acid has a negative effect on health [18]. Grasshoppers have the lowest palmitic acid content, so it can be assumed that they can be a healthier food alternative than other insects, although the content of fatty acids from C4:0 to C13:0, C20:2 to C20:5, and C22:6 occur in low amounts in the range of about 1–8% of the tested fatty acids.

The levels of myristic acid (C14:0) were similar for three of the insects (buffalo worms, house crickets, and grasshoppers), ranging from 0.11 to 0.60, while mealworms had significantly (*p* < 0.05) higher levels of this acid (4.11 g/100 g d.m.). In contrast, grasshopper insects contained high levels of C21:0 (3.69 g/100 g d.m.) and behenic acid (1.99 g/100 g d.m.).

The study indicates a high PUFA/SFA ratio in the mealworm insect species and buffalo worms, which are approximately 2.35 and 0.53, respectively. Mealworms have a four times higher PUFA/SFA content than buffalo worms. House crickets and grasshoppers are characterized by a ratio of approximately 0.22–0.36 PUFA/SFA, which is below the recommended dietary intake of 0.40. FAs, also present in the insects studied, are the dietary factor with the greatest negative impact on LDL cholesterol [18]. The highest content of SFAs is found in grasshoppers at 27.63 g/100 g d.m., and the lowest in house crickets at 7.18 g/100 g d.m. Mealworms and buffalo worms had a slightly higher SFA content of about 2–7 g more. On the other hand, they showed a high PUFA/SFA ratio. The PUFA and MUFA of the n-6 family have been shown to effectively reduce plasma cholesterol concentrations [16,18]. Mealworms (32.78 g/100 g d.m.) have the highest PUFA content, followed by grasshoppers (9.91 g/100 g d.m.), then buffalo worms (4.64 g/100 g d.m.) and house crickets (1.61 g/100 g d.m.). The content of MUFA as well as PUFA also showed an incredibly low content in the house crickets, which correlates with the low content of PUFA/SFA. House crickets contained higher amounts of MUFA than PUFA [18].

Other research has shown that the lipid content was similar in all insect species, and that C16:0 was the major saturated fatty acid. C18:1n9 and C18:2n6 were the most abundant monounsaturated and polyunsaturated fatty acids, respectively, which confirms the results of our study [19]. What is important is the n-6 family was much higher than the n-3 in the PUFA fractions [19]. For comparison, the tested insects had a higher content of n-6/n-3 fatty acids than animal products [18,19]. The highest values were in mealworms and grasshoppers, followed by buffalo worms and house crickets. Oonincx et al. [16], in their research, also show higher content of PUFA in relation to MUFA [16].

The effect of omega-3/6 fatty acids on the prevention of Alzheimer’s disease is widely described in the literature. The reduced content of n-3 and n-6 acids occurs in the plasma and the membrane of erythrocytes in people suffering from neurodegenerative disorders [20,21]. Omega-3 acids (docosahexaenoic acid (DHA) and eicosapentaenoic acid (EPA)) are structural components within the nervous tissue; they increase cognitive performance and reduce synaptic decyphyte and oxidative and amyloid damage [22,23,24]. A literature review by Thomas et al. indicates DHA and EPA supplementation for the prevention of the progression of AD in its initial stages [20]. The use of 240 mg DHA/240 mg EPA supplementation daily for over 12 months positively improves memory in people with early-stage AD. The use of shorter therapy did not improve cognitive functions [20]. According to research by Husain et al. [25], a diet rich in n-3 fatty acids has a significant effect on the hippocampus. However, the effect of these acids also depends on the carrying of the ε4 allele (APOE4), as well as whether the patient has omega-3 metabolism disorders [25]. In the conducted study, DHA acid was present only in mealworms in the amount of 80 mg/100 g; in other tested edible insects, this acid was not detected (Table 1). The demand for DHA in a woman’s diet is 100 mg, while for men, the demand is 120 mg; therefore we can assume that consuming 150 g of mealworms in the daily diet will cover the demand for this acid [26]. EPA acid is present in two tested insects: mealworms (20 mg/100 g) and house crickets (60 mg/100 g). The consumption of EPA in the daily diet is not less than 210 mg per day [26]. Alpha linolenic acid (ALA, C18:3) is found below 1 g/100 g in edible insects; only mealworms contained more than 1 g at 1.64 g/100 g (Table 1). The consumption of ALA in the daily diet increases the content of EPA/DHA in human plasma. ALA is converted into EPA (0.2–21%), less often into DHA (0–9%) [20,27,28]. Both LA and ALA affect the mechanisms regulating the immune functions of microglia through modulating the immune response of the microglia. A study by Lowry [29] shows that LA reduces the secretion of nitric oxide and induces its synthesis, while ALA lowers only nitric oxide. They may be potential compounds regulating neuroimmune responses, including in AD disease [29].

Linoleic acid (C18:2, LA, n-6) occurs in each of the insects, especially mealworms (30.22 g/100 g), and can be a valuable source of inhibiting proinflammatory enzymes in AD disease. LA is a potential compound involved in the inhibition of BACE1. According to Lee et al. [30], disturbed calcium homeostasis can cause μ-calpain hyperactivation. This results in the accumulation of β-amyloid peptides and an increase in BACE1 levels. Researchers demonstrated how conjugated linoleic acid inhibited μ-calpain (IC50 = 2.14 ± 0.44 μM), while LA alone inhibited it at a concentration of 32.66 μM; cathepsin B, 6.22 ± 2.62 μM; and cathepsin L, 4.04 ± 3.24 µM. LA and conjugated LA with calcium has a potential neuroprotective effect [30]. Young et al. [31] evaluated the inhibitory activity of the beta-site APP cleaving enzyme 1 (BACE1) enzyme using *T. molitor* larvae and its major compounds, including LA and oleic acid [31]. They showed high inhibition of *T. molitor* using LA extracts and oleic acid, where the maximum inhibitory concentration was demonstrated by oleic acid (IC50—61.31 μM) [31]. The ratio of n-6/n-3 fatty acids in the human diet is especially important. The correct ratio of n-6/n-3 fatty acids affects the proper functioning of the hippocampus [32,33].

To compare the quality of lipids, IA, IT, and HH indices were used. The index of atherogenicity (IA) values ranged from 0.07 to 0.92. The highest IA for house crickets is 0.92, then two times less for mealworms (0.32), then the lowest for buffalo worms (0.16) and grasshoppers (0.07), which corresponds to the value in commercial fish or chicken fat [34,35]. It suggests that the consumption of insects will not promote the formation of blood clots in blood vessels. The highest index values of IT and IA characterized house crickets.

The remaining insects were characterized by incredibly low values of these coefficients. HH (proportion of hypo- and hypercholesterolemic acids) values ranged from 1.05 to 8.50, respectively, the lowest for house crickets and the highest for grasshoppers. From the nutritional point of view, the highest proportion of HH is found in grasshoppers and mealworms (Table 1) [36,37]. The fatty acids of insects are comparable to those of fish or poultry in their degree of unsaturation and more PUFA [38]. For mealworms and buffalo worms, the HH values are comparable to approx. six.

The analysis of fatty acids suggests that edible insects, and in particular mealworms, are an important source of fatty acids in the prevention of Alzheimer’s disease; considering the increasing pollution of water and air, they may be an alternative food product to fish in the future

### 2.2. Total Phenolic Compounds of an Edible Insect

The amount of TPC in insects varies and depends not only on the sum of polyphenol and flavonoids, but also tannins and other ingredients that may affect the final effect [39,40,41]. Several previous studies have documented the presence of phenolic compounds in the cuticle or secretions from the defensive glands of insects [42,43].

**Figure 1:**
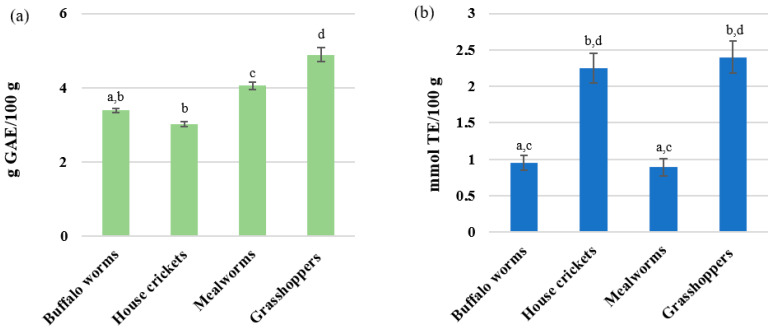
Total polyphenol content (**a**) and antioxidant activity (**b**). Data are expressed as the mean ± SD (*n* = 4). Data marked with different lowercase letters are significantly different (*p* < 0.05).

As shown in Figure 1a, variable values of TPC were obtained for all the insects, which were within the range of 3.02–4.89 g GAE/100 g d.m. The highest content of polyphenols was found in grasshoppers with a value of 4.89 ± 0.21 g GAE/100 g d.m. and mealworms with the value 4.05 ± 0.22 g GAE/100 g d.m. The lowest polyphenol content was found in house crickets, with 3.02 g GAE/100 g d.m., followed by buffalo worms, with 3.39 g GAE/100 g d.m. Grasshoppers and mealworms differed significantly (*p* < 0.05). Del Hierro et al. [40], in their research on the content of TPC, demonstrated that the content of TPC in yellow mealworm and house cricket extracts (ethanol or/and water) is within the range of 0.3–5 g GAE/100 g of extract. These results suggest that the type and process of preparation significantly affect the content of polyphenols in insects. Additionally, an important fact is that a freshly consumed insect may contain more polyphenols than a processed one [40]. This is also supported by Liu [44], who describes that catechin is responsible for the high difference in TPC in insects. The change in the content of polyphenols could be due to the varied diet of the insects analyzed or from the chitin content of the cuticle [44]. Edible insects contain, among others, 4-hydroxybenzoic acid, p-coumaric acid, ferulic acid, and syringic acid [39,40,41,42,43,44,45]. They are metabolized in the digestive system from consumed plants. During digestion, the digestive system delicately absorbs compounds such as flavonoids, e.g., quercetin, flavones, and phenolic acids, such as sinapic acid [41,45]. However, there are also phenolic compounds not related to the diet of insects, because they are synthesized by phenolic oxidase from the epidermis of insects [45]. These compounds have antioxidant properties, and therefore diets rich in antioxidants can reduce oxidative stress in people with AD [46,47,48,49,50]. Phenolic compounds counteract AD through inactivating free radicals, inhibiting pro-inflammatory enzymes, regulating intracellular pathways and signals, and interacting with transition metals [46]. In the central nervous system, phenolic compounds may counter chronic inflammation [49,50].

The cooked edible beetle *Eulepida mashona* contains TPC of 0.81 mg GAE/1 g of dry weight, while ground cricket contains 7.7 mg GAE/g [45]. It can be concluded that the freeze-drying process retains a higher TPC content that is several times greater than in the cooking process [35].

Regardless of the source, however, TPC in edible insects is high. Daily eating insects, especially grasshoppers and mealworms, can positively affect the body.

### 2.3. Antioxidant Activity of an Edible Insect

The highest antiradical activity against DPPH• was noted for mealworms (IC50 value 87.95 μg/mL) as well as buffalo worms (IC50 value 95.59 μg/mL), (*p*  <  0.05) (Figure 1b). House crickets and grasshoppers have a high antioxidant potential, with IC50 = 125.00–180.00 μg/mL, respectively (*p* < 0.05). The antioxidant activity is not correlated with TPC, which is related to the fact that the TPC method shows not only phenolic compounds, but also other compounds, such as tannins, etc., (Figure 1a,b). The insects retain high-level properties of antiradical activity under free-dried processing conditions. There is little research on the antioxidant properties of freeze-dried edible insects. Freeze-drying is among the key processes that can have a significant impact not only on the polyphenol antioxidant activity, but also other nutrients found in the tested insects. According to previous findings in the literature, it is relevant that Hys, Pro, Tyr, and peptides (low molecular weight) have higher antioxidant activities [51,52,53]. Kim et al. [54] demonstrated the antioxidant effects of mealworm extract scavenging activity of 91.8 μg/mL based on IC50. The results of the above-mentioned study revealed a 4% higher antioxidant activity (lower IC50) in the mealworm. The difference is probably due to the solvents used [54]. Baek et al. [55] showed that the scavenging activities of mealworms cooked via various methods is in the range of 2000 μg/mL [55]. Lyophilization brought a better scavenging effect by about 23 times and about 10 times considering the water content of the processed insects [55]. 

The studies showed that the IC50 of the tested samples of house crickets is comparable to the data in the literature. The differences are due to the difference in how the insects were processed. According to Ugur et al. [56], extraction efficiency increases with temperature, and that provided higher recovery of antioxidant compound oils in cricket in the range of 0.472–0.647 mg Trolox/g oil [56]. According to Chatsuwan et al. [57], the antiradical activities of grasshoppers were of 204.67 and 176.31 µg/mL (IC50) [57], which correlates with the presented research results.

Oxidative stress is a crucial factor in developing AD. It leads to inflammation; DNA, protein, and lipid oxidation; cognitive decline; and neurodegenerative disorders [58,59,60]. The lipids, vitamins, and phenolic compounds contained in the tested insects may play a key role in combating the pathogenesis of AD. Mealworms have the highest antioxidant potential and may be a useful source of antioxidants in the prevention of AD.

### 2.4. The content of the Vitamins of an Edible Insect

Edible insects have a high vitamin content. The different content of vitamins depends on the diet of the insects. The study examined vitamins B, C and E; the results are presented in Table 2.

The highest content of B vitamins was found in house crickets in the range of 7.27 mg/100 g; then mealworms, amounting to 6.77 mg/100 g; and grasshoppers, being 5.27 mg/100 g. Buffalo worms had the lowest vitamin B content of 1.51 mg/100 g. The daily requirement of vitamin B1 for women is 1.1 mg/day, and for men, 1.2 mg/day [61]. Consuming 100 g of grasshoppers will satisfy 43%, or in the case of mealworms, approximately 19%, of the daily requirement for this vitamin. In the case of vitamin B2, the requirement for women (1.1 mg/day) and men (1.3 mg/day) [61] can be provided through the consumption of edible food in the amount of about 33 g mealworms/day or 41–42 g house crickets or grasshoppers/day. In the case of buffalo worms, women should consume 100 g, and men should consume slightly more than 100 g. In the case of vitamin B3, the consumption of 100 g/day of edible insects in the form of mealworms meets the requirement of 25–29% (for men, 16 mg/day; women, 14 mg/day [61]); in the case of house crickets and grasshoppers, it ranges from 19 to 23%; for buffalo worms, it is less than 8%. On the other hand, the requirement for vitamin B6 is 1.7 mg/day for men and 1.5 mg/day for women [61]. When consuming insects in the amount of 100 g, 46% of the daily requirement will be covered by mealworms, then about 12% by house crickets and grasshoppers, while buffalo worms are below 5%. The daily requirement of vitamin B9 for both men and women is 0.4 mg/day [62]. The consumption of 50 g grasshoppers in the daily diet will meet the daily requirement of vitamin B9. For mealworms, required consumption is 174 g, and house crickets and buffalo worms would need over 230 g. Insects are low in vitamin C; a daily intake of 100 g of insects will take up 10% of the daily requirement for grasshoppers, 2% for mealworms or house crickets, and less than 1% for buffalo worms. Vitamin E is the highest in mealworms and house crickets, amounting to 0.98 IU and 0.79 IU, respectively. AD is multi-level, so therapies to help prevent AD need a variety of ingredients. Vitamins are such ingredients that, in addition to a few functions, also exhibit antioxidant activity [63]. B vitamins support the prevention of cognitive disorders through providing methyl groups, increasing DNA methylation. Vitamin B6, B9, and B12 are co-factors of the homocysteine-to-methionine conversion reaction. On the other hand, lowering the content of vitamin B9 disturbs the metabolism of acetylcholine [63,64,65]. A diet rich in vitamins C and E also improves cognitive performance in people with AD through its neuroprotective effects. Vitamin E reduces lipid peroxidation and reduces the deposition of β-amyloid; vitamin C has a similar effect [63].

Edible insects are characterized by a high content of micro- and macroelements, but also a high content of vitamins, 3–4 times higher than in chicken meat or beef. Meat offal, which is not popular, contains a higher content of unfavorable fatty acids [66].

### 2.5. Isothermal Titration Calorimetry of an Edible Insect

ITC is a sensitive method showing the exact thermodynamic properties of protein ligand binding. ΔH is negative for all cases, which means that the reaction is exothermic. With the reaction time, the available bonding sites in extracts of edible insects decreased until it finally saturated. The thermodynamic parameter data are listed in Table 3.

ITC is a sensitive method showing the exact thermodynamic properties of protein ligand binding. ΔH is negative for all cases, which means that the reaction is exothermic. With the reaction time, the available bonding sites in extracts of edible insects decreased until it finally saturated. Edible insect extracts in their composition are characterized by a high content of B vitamins, omega-9 fatty acids, but also phenolic compounds derived from plants consumed with the diet. The thermodynamic parameter data are listed in Table 3. The binding constants were obtained through experiments. Extracts of edible insects can bind to AChE. The highest association constant (KA) was found in the mealworm extract, followed by house crickets, grasshoppers, and the lowest in buffalo worms (Table 3). ΔH ranged from −11.22 to 93.98 kJ/mol for buffalo worms and mealworms, respectively, which strongly correlates with KA. On the other hand, the highest affinity for AChE was shown by mealworms, amounting to −40.75 kJ/mol, followed by grasshoppers at −33.69 kJ/mol; a slightly lower affinity was shown by house crickets at −31.16 kJ/mol, and the lowest affinity was for buffalo worms, amounting to −17.22 kJ/mol. It was observed that the most consistent complexes and the highest affinity with AChE were found in extracts from only three insects: house crickets, mealworms, and grasshoppers. The highest activity of an AChE inhibitor was demonstrated by mealworms at 94.45%. Buffalo worms extract showed activity that was approx. seven times lower (13.59%). On the other hand, the remaining extracts were characterized by an activity of about 81–83%. The Ki of the mealworm extracts was so low that it was below the calculation range, suggesting high binding affinity in the presence of ACh. However, in the case of grasshoppers extracts, Ki was 0.41 μmol/L, while for house crickets, it was 0.54 μmol/L.

### 2.6. The Effect of the Addition of Edible Insects on the Nutritional Value of Olive Oil

Based on the research, we can conclude that the content of omega-3, 6, and 9 fatty acids (Table 4) increased in comparison to olive oil without the addition of insects. For olive oil with grasshopper, they increased by 1.22%, while with the addition of mealworms, they increased by 7.76%. Essential fatty acids were also increased by 13–15% for MUFA and about 1–8% for PUFA. 

TPC also increased by 1.7 times for mealworms and 3.8 times for grasshoppers. The antioxidant potential increased slightly. However, these changes were not statistically significant (*p* > 0.05). In addition, the oil has been naturally enriched with vitamin C and B vitamins. They also slightly increased the content of vitamin E (Table 2). The enrichment of olive oil with vitamins and the higher content of omega-3, 6, and 9 fatty acids resulted in the fact that olive oil with the addition of edible insects showed a higher affinity for the AChE enzyme than the fruit alone or olive oil without additives (Table 3).

## 3. Materials and Methods

### 3.1. Chemicals and Materials

GC/MS-grade heptane, boron trifluoride-methanol solution 14% in methanol, and hexane, in addition to LC/MS-grade methanol, water, acetylthiocholine iodide, acetonitrile, folic acid, thiamine, riboflavin, ultrapure water, pyridoxine, niacin, Acetylcholinesterase (from Electrophorus electricus), and 2,2-diphenyl-1-(2,4,6-trinitrophenyl) hydrazyl, were purchased from Merck (Darmstadt, Germany). Fatty acid methyl esters (unsaturated Kit) and sodium chloride were purchased from Sigma-Aldrich (St. Louis, MO, USA). All other reagents were of analytical grade and were purchased from Chempur (Piekary Slaskie, Poland).

Freeze-dried buffalo worms (Alphitobius diaperinus Panzer), house crickets (Gryllus campestris), mealworms (Tenebrio molitor), and grasshoppers (Chorthippus biguttulus) were purchased from a local commercial source (Delibugs, Lelystad, Netherlands). Before analysis, the insects were shredded.

Insects (10 g) previously ground in a mortar were added to virgin olive oil (1 L). Olive oil with insects was left for 48 h in a cool, dark place.

### 3.2. Analysis of the Fat Acid via GC–MS

**Figure 2:**
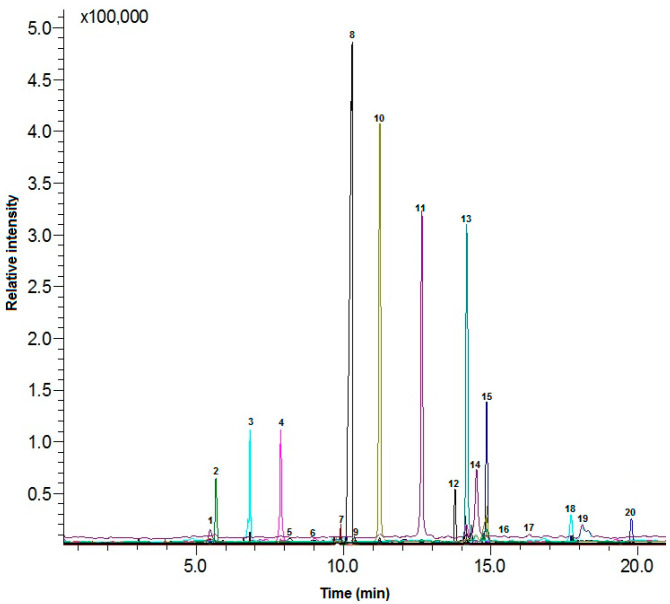
GC/MS chromatogram of fatty acids of Buffalo worms. Peaks: (1) C4:0; (2) C18:3; (3) C20:0; (4) C20:1; (5) C10:0; (6) C8:0; (7) C12:0; (8) C18:1; (9) C6:0; (10) C16:1; (11) C16:0; (12) C:23:0; (13) C18:2; (14) C17:1; (15) C14:0; (16) C18:0; (17) C13:0; (18) C21:0; (19) C17:0; (20) C15:0.

The insects were ground in a mortar, and then an extract (1 g ground insects in 10 mL ethanol) was prepared for analysis and characterized using a Shimadzu GC-MS (Thermo scientific, Waltham, MA, USA) according to Olarte Mantilla et al. [67] with small modifications. Extracts were dissolved (10 mg/mL) in Recti-Therm™ I #TS-18821 and hydrolyzed with methanolic NaOH solution (20 g/L) at 60 °C (1 h) with continuous magnetic stirring. After hydrolysis, a methanolic solution of BF3 was introduced to the reaction vial. The mixture was heated at 60 °C (3 min), and then heptane was added and cooled to 24 °C. A saturated NaCl solution was added, and phase separation was allowed. The samples were then rested at 24 °C for 5 min, after which they were centrifuged for 3 min (5000 rpm) (Centrifuge MPW-260R, MPW MED. Instruments, Warsaw, Poland). The supernatants were analyzed in a Shimadzu GC–MS QP2010 SE (Shimadzu, Tokyo, Japan). The column employed was a Zebron ZB-FFAP UI capillary column: 30 m × 0.250 mm × 0.25 μm. The carrier gas was helium (1 mL/min). An AOC-20i (Shimadzu, Tokyo, Japan) autoinjector was used, with 1 μL injections in spitless mode, and the injector temperature was 250 °C. The oven was initially set at 120 °C and increased by 2 °C/min to 250 °C. The inlet temperatures at the MS were set at 250 °C and those at the MS ion source and the interface were 120 °C and 250 °C, respectively [67]. The scan mass range was 30–600 *m*/*z*. The identification of compounds was performed using commercial standards kits (Figure 2). The fatty acids of the methyl esters were analyzed in samples of olive oil with or without edible insects, which were then subjected to a methylation reaction. Assays were prepared for determination. Approximately 0.01 g of olive oil was weighed into the reaction vials, then proceeded as above.

### 3.3. Analysis of the Nutritional Quality of Lipids

The polyunsaturated/saturated fatty acids (PUFAs/SFAs) were analyzed according to the method described by Santos-Silva et al. [68]. Two indices associated with the risk of coronary artery disease were used, i.e., the thrombogenic index (TI) and the atherogenic index (AI), in addition to the ratio of hypocholesterolemic and hypercholesterolemic fatty acids (HH). AI = (C12:0 + 4 × C14:0 + C16:0)/(MUFA + n-6 + n-3); TI = (C14:0 + C16:0 + C18:0)/(0.5 × MUFA + 0.5 × n-6 + 3 × n-3 + n-3/n-6); HH= (C18:1 c9 + C18:2 n-6 + C18:3 n-3 + C20:3 n-6 + C20:4 n-6 + C20:5 n-3 + C22:5 n-3)/(C12:0 + C14:0 + C16:0) [68].

### 3.4. Total Phenolics Content (TPC)

The content of total phenolic compounds (TPC) of the extracts was determined via Folin–Ciocalteu (FC) assay according to a modified procedure from Di Mattia et al. [69] with some modifications. The edible worms powder (0.5 g) was taken into a flask (50 mL) with deionized water (20 mL) and, next, was shaken for 1 h and then filtered. Next, 0.1 mL of extracts at 4.9 deionized water were mixed with 500 μL of FC reagent. After 3 min, 1.5 mL of a 25% Na_2_CO_3_ solution was added, followed by deionized water and incubation (1 h). The absorbance at 765 nm was measured. The results were expressed as gallic acid equivalents (GAE) [g/100 g] (12.5 to 1000 μg/mL) [69]. Olive oil samples with and without edible insects were weighed out at 20 g and dissolved in 100 mL of hexane. Then, the polyphenols were extracted through adding a mixture of water and methanol in the amount of 40:60 *v*/*v* and shaking for 5 min, and then the water and alcohol fractions were separated on a separatory funnel. A total of 0.1 mL was taken from the aqueous fraction of the extract, and the method described above was followed.

### 3.5. DPPH•

The radical scavenging activity (DPPH•) was determined according to Zielińska et al. [70] with small modifications. The edible worm powder (1 g) and 70% methanol (20 mL) were added to flask and shaken (1 h). Next, a 0.25–0.50 mL (filtrated) volume of the sample was mixed with 1.75–1.95 mL of a 0.50 mL solution of DPPH in 70% methanol and incubated (24 °C at 0.5 h). The absorbance was measured at 517 nm. The scavenging effect was calculated according to the following equation: Antioxidant activity (%) = [AControl − AExtract)/AControl] × 100 [70]. Olive oil samples with and without edible insects were weighed out at 20 g and dissolved in 100 mL of hexane. Then, the antioxidants were extracted through adding a mixture of water and methanol in the amount of 40:60 *v*/*v* and shaking for 5 min, and then the water and alcohol fractions were separated on a separatory funnel. Next, 0.25–0.50 mL was taken from the aqueous fraction of the extract, and the method described above was followed. 

### 3.6. Vitamins B, C, and E Profile According to LC-ESI-MS

**Figure 3:**
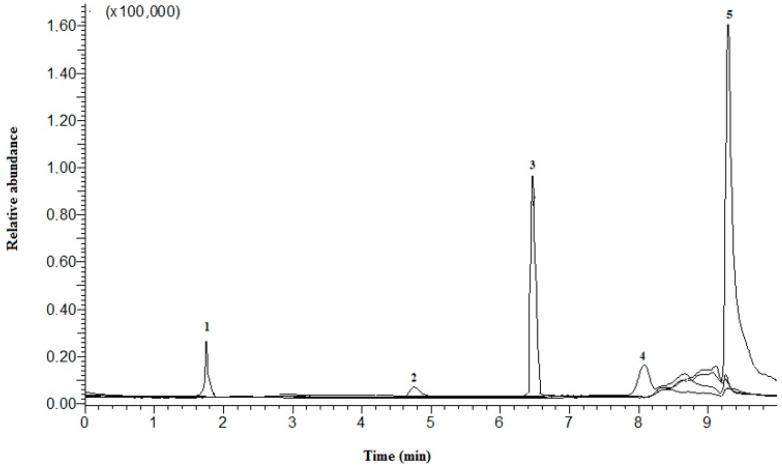
LC–MS chromatograms of vitamins B of house crickets. Peaks: (1) folic acid; (2) thiamine; (3) riboflavin; (4) pyridoxine; (5) niacin.

Buffalo worm, house cricket, mealworm, and grasshopper extracts (10 mg/mL) were dissolved in 0.1% (*v*/*v*) formic acid in water and filtered using a 0.2 μm, 17 mm nylon filter. The analysis was performed by means of the UHPLC-ESI-MS liquid chromatography method using a UHPLC chromatograph with an ESI-MS detector (Shimadzu, Kyoto, Japan). The chromatographic analysis system consisted of a CBM-20A controller, two LC-2020AD pumps, an automatic SIL-30AC sampler, and a CTO-20AC column oven, equipped with a photodiode array detector and a mass spectrometer (LCMS-2020) with an electrospray ionization source—ESI (LCMS-2020, Shimadzu, Tokyo, Japan). Then, 2 μL samples were injected onto a Kinetex C18 column (2.1 × 150 mm, Thermo Scientific 5 μM particle size). The mobile phases were eluent A, acetonitrile/formic acid (99.9/0.1, *v*/*v*), and eluent B, along with water. The flow rate was 0.2 mL/min, and the gradient was 0–5.00 min, 0–20% B, 5.00–5.20 min, 20–100%; 20–6.20 min, 100–100% B; 6.20–6.40 min, 100–0% B; 6.40–10.00 min, 0%. ESI source was in positive ion mode. The column temperature was 35 °C, drying gas temperature was 250 °C, and capillary temperature was 400 °C. Nitrogen was used as nebulizer gas. Full mass spectra were obtained in the mass range from *m*/*z* 50 to 600 in positive ion mode. The obtained MS spectrum signal data was collected and processed with LabSolutions software. Simultaneously, a standard curve of vitamins thiamine (B1), riboflavin (B2), niacin (B3), pyridoxine (B6), and folic acid (B9) was prepared. The calibration curves were constructed for standard compounds using six different concentration levels in a range of 0.01–1.0 mg/mL [71] (Figure 3).

The extraction of vitamins from olive oil with and without edible insects was carried out with a 1 g sample, extracting with 10 mL hexane and mixing via vortexing. Then, methanol and water were added to the solution in a ratio of 80:20 *v*/*v*, followed by centrifugation at 3000 rpm. The phases were separated in a separatory funnel. The hexane was evaporated. Analyses were performed as above.

Vitamin C—2 μL samples were injected onto a Kinetex C18 column (2.1 × 150 mm, particle size 5 μM; Thermo Scientific. The mobile phases were eluent A, methanol/formic acid (99.9/0.1, *v*/*v*), and eluent B, water/formic acid (99.9/0.1, *v*/*v*). The flow rate was 0.2 mL/min and the gradient was 0–5.00 min, 30–100% B, 5.00–5.01 min, 100% B, 5.01–7.00 min, 100–30% B. ESI source operated in negative ion mode, capillary voltage was set at 4.5 kV, drying gas temperature—250 °C, drying gas flow—15.0 L/min, atomizing gas pressure—1 bar, capillary temperature—350 °C. Nitrogen was used as a nebulizer and the column temperature was 30 °C. Obtained data of UV absorbance signal and MS spectrum were collected and processed using LabSolutions software. Full mass spectra were obtained in the *m*/*z* range from 50 to 350 in positive ion mode.

Calibration curves were made using standard compound standards using six different concentration levels ranging from 0.03–1.00 mg/mL.

Vitamin E—10 μL samples were injected onto a Kinetex C18 column (2.1 × 150 mm, particle size 5 μM; Thermo Scientific). The mobile phases were eluent A, water/formic acid (99.9/0.1, *v*/*v*), and eluent B, methanol/formic acid (99.9/0.1, *v*/*v*) The flow rate was 0.2 mL/min and the gradient was 0–1.00 min, 0% B, 1.00–6.01 min, 0–100% B, 6.01–25.00 min, 100–0% B. ESI source operated in negative ion mode, capillary voltage was set at 4.5 kV, drying gas temperature—250 °C, drying gas flow—15.0 L/min, atomizing gas pressure—1 bar, capillary temperature—350 °C. Nitrogen was used as a nebulizer and the column temperature was 30 °C. Obtained data of UV absorbance signal and MS spectrum were collected and processed using LabSolutions software. Full mass spectra were obtained in the *m*/*z* range from 50 to 500 in positive ion mode.

Calibration curves were made using standard compound standards using six different concentration levels ranging from 0.05–1.00 mg/mL.

### 3.7. Isothermal Titration Calorimetry (ITC) Measurements

**Figure 4:**
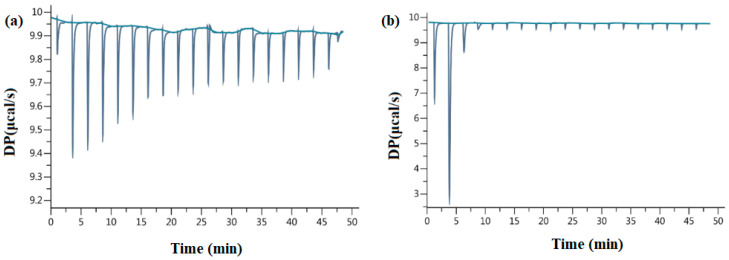
ITC raw data (**a**) AChE interactions and mealworms extract; (**b**) ACh hydrolysis catalyzed by AChE in the presence of mealworms extract.

Calorimetric measurements were performed via isothermal titration calorimetry (ITC) using a MicroCal PEAQ-ITC200 apparatus (Malvern, Worcestershire, UK). The analysis was performed according to Budryn et al.’s [72] procedure with some modifications. The measuring cell of the 0.2 mL calorimeter was filled with degassed AChE solution (20 µmol/L, enzyme diluted with methanol). The syringe of the device was filled with methanol solutions of edible insects: buffalo worms, house crickets, mealworms, grasshoppers, and olive oil with edible insects with with a concentration of 5 mmol/L and/or ACh at a concentration of 5 mmol/L. Measurements: 36.6 °C with constant stirring (307 rpm). Data were calculated using MicroCal PEAQ-ITC Analysis software. To calculate the inhibition of enzyme activity, the signals of ACh hydrolysis in the presence of inhibitors were subtracted from the signals of ACh hydrolysis without inhibitors, considering the enthalpy recorded during the interaction of inhibitors with ACh. The inhibitory activity was calculated as [(∆H − ∆Hi)/∆H] × 100%, where ∆Hi is the signal of ACh hydrolysis in the presence of the inhibitor (minus the signal of the inhibitor-enzyme interaction), and ∆H is the signal of ACh hydrolysis without the inhibitor. The percent inhibition of ACh hydrolysis at a given ligand concentration was calculated and used to calculate the ligand concentration that inhibited the hydrolysis reaction by 50% (IC50) [72]. Figure 4 shows examples of recorded energy effects during the ITC analysis.

### 3.8. Statistical Analysis

Statistical analyses were expressed as the mean value ± standard deviation (SD). Analysis of variance (ANOVA) and the Tukey post hoc tests were applied to determine differences between means. Differences were significant at *p* < 0.05.

## 4. Conclusions

This research showed that edible insects have a high content of fatty acids, phenolic compounds, and vitamins. This makes them a rich source of antioxidants. Another important feature is that they have a high content of good fatty acids, in particular, the n-3 to n-6 ratio and PUFA/SFA. It indicates this that edible insects are the key to solving the growing need for nutrients in the prevention of AD disease. Interestingly, in terms of all factors evaluated, mealworms and grasshoppers turned out to be the most nutritionally valuable edible insects. In contrast, buffalo worms had the least nutritional content. The tested ITC edible insects, in addition to buffalo worms, showed high affinity for AChE. The selection of grasshoppers and powdery mildew larvae improved the nutritional value of olive oil and increased the affinity for AChE. It can therefore be concluded that edible insects, particularly mealworms and grasshoppers eaten in the form of olive oil with added edible insects, can be eaten as functional food. Olive oil may be a potential AChE inhibitor and may improve cognitive function. The research shows the prospect of further research.

## Figures and Tables

**Table 1:** Fatty acids content of edible insects (g/100 g d.m.).

	Buffalo Worms	House Crickets	Mealworms	Grasshoppers
C4:0	0.05 ± 0.01 ^a^	0.003 ± 0.00 ^b^	0.001 ± 0.00 ^b^	0.04 ± 0.00 ^a^
C6:0	0.02 ± 0.00 ^a^	0.01 ± 0.00 ^a^	0.001 ± 0.00 ^b^	0.02 ± 0.00 ^a^
C8:0	0.01 ± 0.00 ^a^	0.01 ± 0.00 ^a^	0.002 ± 0.00 ^b^	0.19 ± 0.00 ^c^
C10:0	0.02 ± 0.00 ^a^	0.01 ± 0.00 ^a^	0.02 ± 0.00 ^a^	0.03 ± 0.00 ^a^
C12:0	0.03 ± 0.01 ^a^	0.03 ± 0.00 ^b^	0.35 ± 0.01 ^c^	0.04 ± 0.00 ^a^
C13:0	0.01 ± 0.01 ^a^	0.002 ± 0.00 ^b^	0.15 ± 0.04 ^c^	0.01 ± 0.00 ^a^
C14:0	0.60 ± 0.09 ^c^	0.11 ± 0.01 ^c^	4.11 ± 0.02 ^d^	0.19 ± 0.01 ^c^
C15:0	0.12 ± 0.02 ^c^	0.01 ± 0.00 ^a^	0.14 ± 0.00 ^c^	0.63 ± 0.03 ^c^
C16:0	5.85 ± 0.09 ^k,f^	4.84 ± 0.15 ^e^	5.45 ± 0.32 ^k,f^	3.46 ± 0.45 ^k^
C17:0	0.09 ± 0.01 ^a^	0.08 ± 0.00 ^a^	0.19 ± 0.00 ^c^	0.49 ± 0.05 ^c^
C18:0	0.01 ± 0.00 ^a^	0.01 ± 0.00 ^a^	0.01 ± 0.00 ^a^	0.004 ± 0.00 ^b^
C20:0	0.42 ± 0.01 ^c^	0.14 ± 0.02 ^c^	0.19 ± 0.01 ^c^	0.02 ± 0.00 ^a^
C21:0	0.08 ± 0. 00 ^a^	0.004 ± 0.00 ^b^	nd	3.69 ± 0.05 ^d^
C22:0	nd	0.07 ± 0.00 ^a^	0.03 ± 0.00 ^a^	1.99 ± 0.09 ^d^
C23:0	0.23 ± 0.02 ^c^	nd	nd	0.11 ± 0.01 ^c^
C24:0	nd	0.03 ± 0.00 ^a^	nd	nd
C16:1	10.44 ± 0.39 ^k^	0.16 ± 0.02 ^a^	1.49 ± 0.15 ^a^	22.02 ± 0.05 ^l^
C17:1	0.35 ± 0.02 ^a^	0.02 ± 0.00 ^a^	0.03 ± 0.00 ^a^	0.05 ± 0.00 ^a^
C18:1	33.13 ± 2.45 ^g^	3.94 ± 0.39 ^d^	37.36 ± 1.10 ^h^	30.00 ± 0.09 ^h^
C20:1	0.43 ± 0.02 ^c^	0.03 ± 0.00 ^a^	0.15 ± 0.00 ^c,i^	0.15 ± 0.00 ^c,i^
C22:1	nd	0.15 ± 0.01 ^c,i^	nd	nd
C18:2	4.64 ± 0.02 ^d^	1.18 ± 0.05 ^d^	30.22 ± 2.22 ^h^	5.02 ± 0.15 ^e^
C18:3	0.29 ± 0.01 ^c^	0.12 ± 0.00 ^c^	1.64 ± 0.45 ^c^	0.19 ± 0.00 ^c^
C20:2	nd	0.20 ± 0.01 ^c^	0.12 ± 0.01 ^a^	0.31 ± 0.01 ^c,j^
C20:3	nd	0.45 ± 0.01 ^c^	0.01 ± 0.00 ^a^	0.33 ± 0.01 ^c,j^
C20:4	nd	nd	0.10 ± 0.00 ^c^	0.77 ± 0.02 ^c^
C20:5	nd	0.06 ± 0.00 ^a^	0.02 ± 0.00 ^a^	nd
C22:6	nd	nd	0.08 ± 0.00 ^a^	nd
SFA	9.34 ± 0.15 ^l^	7.18 ± 1.15 ^d^	13.94 ± 0.49 ^l^	27.63 ± 1.09 ^h^
PUFA	4.95 ± 0.02 ^d^	1.61 ± 0.09 ^n^	32,78 ± 0.35 ^h^	9.91 ± 0.02 ^e^
MUFA	44.35 ± 1.05 ^m^	4.16 ± 0.45 ^d^	39.03 ± 0.25 ^h^	52.22 ± 0.02 ^g^
PUFA/SFA	0.53 ± 0.02 ^c^	0.22 ± 0.0 ^c^	2.35 ± 0.15 ^n^	0.36 ± 0.01 ^a^
PUFA n-3	0.31 ± 0.02 ^c^	0.44 ± 0.02 ^c^	2.45 ± 0.25 ^d^	3.79 ± 0.02 ^d^
PUFA n-6	4.64 ± 0.09 ^d^	1.18 ± 0.03 ^n^	30.33 ± 0.33 ^h^	6.12 ± 0.45 ^a^
PUFA n-6/PUFA n-3	14.97 ± 0.25 ^k^	2.69 ± 0.02 ^n,d^	12.38 ± 0.02 ^k^	1.62 ± 0.01 ^n^
AI	0.16 ± 0.02 ^c^	0.92 ± 0.02 ^n^	0.31 ± 0.02 ^c^	0.07 ± 0.01 ^c^
TI	0.32 ± 0.02 ^c^	1.56 ± 0.02 ^n^	0.31 ± 0.01 ^c^	0.49 ± 0.01 ^c^
HH	5.83 ± 0.09 ^d^	1.05 ± 0.02 ^c^	6.84 ± 0.02 ^n,d^	8.50 ± 0.02 ^n,d^

Values are expressed as mean value ± SD; *n* = 4. ^a–n^ Different letters in one row correspond to significant differences (*p* < 0.05); nd—not detected.

**Table 2:** Vitamin content of edible insects and olive oil with/without of edible insects.

Vitamin	Buffalo Worms	House Crickets	Mealworms	Grasshoppers	Olive Oil	Olive Oil with Grasshoppers	Olive Oil with Mealworms
B_1_ mg/100 g	0.01 ± 0.00 ^a^	0.05 ± 0.00 ^a^	0.23 ± 0.02 ^b^	0.52 ± 0.01 ^c^	nd	0.22 ± 0.01 ^c^	0.09 ± 0.0 ^b^
B_2_ mg/100 g	0.22 ± 0.01 ^a^	3.65 ± 0.29 ^b^	1.44 ± 0.09 ^b^	0.69 ± 0.01 ^c^	nd	0.36 ± 0.01 ^c^	0.74 ± 0.03 ^e^
B_3_ mg/100 g	1.18 ± 0.15 ^a^	3.19 ± 0.13 ^b^	4.08 ± 0.59 ^c^	3.08 ± 0.19 ^b^	nd	1.15 ± 0.02 ^a^	1.89 ± 0.05 ^b^
B_6_ mg/100 g	0.08 ± 0.01 ^a^	0.21 ± 0.01 ^a^	0.79 ± 0.02 ^b^	0.19 ± 0.00 ^a^	nd	nd	0.39 ± 0.00 ^b^
B_9_ mg/100 g	0.02 ± 0.00 ^a^	0.17 ± 0.02 ^a^	0.23 ± 0.01 ^b^	0.79 ± 0.09 ^c^	nd	0.31 ± 0.00 ^c^	0.05 ± 0.00 ^b^
The total content of B vitamins	1.51 ± 0.03 ^a^	7.27 ± 0.15 ^b^	6.77 ± 0.23 ^b^	5.27 ± 0.03 ^c^	nd	2.04 ± 0.01 ^a^	3.16 ± 0.10 ^a^
Vitamin C mg/100 g	0.96 ± 0.01 ^a^	2.18 ± 0.02 ^b^	2.25 ± 0.03 ^b^	8.15 ± 0.15 ^c^	nd	3.12 ± 0.03 ^c^	0.65 ± 0.00 ^b^
E (IU)	0.01 ± 0.00 ^a^	0.79 ± 0.01 ^b^	0.98 ± 0.02 ^b^	0.29 ± 0.00 ^a^	14.21 ± 0.01	14.35 ± 0.00 ^c^	14.81 ± 0.09 ^d^

Values are expressed as mean value ± SD; *n* = 4; ^a–e^ different letters in one row correspond to significant differences (*p* < 0.05); nd—not detected.

**Table 3:** The thermodynamic parameters obtained from ITC.

Compound	K_A_ × 10^3^ (L/mol)	ΔH (kJ/mol)	ΔG (kJ/mol)	% Inhibition	IC50 (μmol/μmol AChE)
Buffalo worms	1.02 ± 0.45 ^a^	−11.22 ± 1.32 ^a^	−17.22 ± 3.05 ^a^	13.59 ± 0.13 ^a^	45.79 ± 1.18 ^a^
House crickets	8.19 ± 0.39 ^b^	−63.61 ± 4.35 ^b^	−31.16 ± 2.05 ^b^	83.02 ± 2.95 ^b^	5.35 ± 0.39 ^b^
Mealworms	9.33 ± 0.10 ^b^	−93.98 ± 4.18 ^c^	−40.75 ± 2.45 ^c^	94.45 ± 2.54 ^b^	1.98 ± 0.45 ^c^
Grasshoppers	7.25 ± 0.02 ^b^	−85.11 ± 5.12 ^c^	−33.69 ± 3.39 ^b^	81.65 ± 1.29 ^b^	5.01 ± 0.15 ^b^
Olive oil	5.79 ± 0.05 ^a^	−55.18 ± 1.05 ^b^	−28.28 ± 2.11 ^b^	78.95 ± 0.45 ^b^	7.55 ± 0.02 ^b^
Olive oil with grasshoppers	10.28 ± 0.03 ^b^	−94.95 ± 3.55 ^b^	−45.24 ± 1.15 ^c^	115.12 ± 1.35 ^c^	1.87 ± 0.01 ^b^
Olive oil with mealworms	33.60 ± 0.15 ^d^	−115.45 ± 4.45 ^c^	−48.33 ± 2.69 ^c^	125.29 ± 1.45 ^c^	1.95 ± 0.03 ^c^

Values are expressed as mean value ± SD; *n* = 4; ^a–d^ Different letters in one row correspond to significant differences (*p* < 0.05).

**Table 4:** Content of fatty acids, total polyphenol content (TPC), and antioxidant activity (DPPH) in olive oil without and from edible insects.

	Olive Oil	Olive Oil with Grasshoppers	Olive Oil with Mealworms
C14:0 [%]	0.01 ± 0.00 ^a^	0.03 ± 0.00 ^a^	0.22 ± 0.01 ^b^
C16:0 [%]	12.45 ± 0.05 ^a^	13.33 ± 0.15 ^a^	14.23 ± 0.10 ^b^
C16:1 n-7 [%]	0.95 ± 0.01 ^a^	6.34 ± 0.03 ^b^	0.98 ± 0.00 ^a^
C18:0 [%]	2.55 ± 0.02 ^a^	2.55 ± 0.01 ^a^	2.55 ± 0.00 ^a^
C18:1 n-9 [%]	72.85 ± 0.15 ^a^	81.94 ± 0.39 ^b^	85.06 ± 0.45 ^b^
C18:2 n-6 [%]	9.88 ± 0.01 ^a^	11.06 ± 0.15 ^b^	17.35 ± 0.39 ^c^
C18:3 n-3 [%]	0.85 ± 0.00 ^a^	0.89 ± 0.01 ^a^	1.14 ± 0.15 ^b^
C20:0 [%]	0.46 ± 0.00 ^a^	0.46 ± 0.00 ^a^	0.52 ± 0.01 ^b^
ΣMUFA (n-9, n-7) [%]	73.80 ± 0.03 ^a^	88.28 ± 0.15 ^b^	86.04 ± 0.68 ^b^
ΣPUFA (n-3, n-6) [%]	10.73 ± 0.05 ^a^	11.95 ± 0.10 ^a^	18.49 ± 0.15 ^b^
ΣUFA [%]	84.53 ± 0.15 ^a^	100.23 ± 0.59 ^b^	104.53 ± 0.98 ^b^
ΣSFA [%]	15.47 ± 0.30 ^a^	16.37 ± 0.15 ^a^	17.52 ± 0.09 ^b^
TPC [g GAE/100 g]	0.67 ± 0.01 ^a^	2.56 ± 0.09 ^b^	1.18 ± 0.09 ^b^
DPPH [mmolTE/100 g]	0.85 ± 0.01 ^a^	0.84 ± 0.00 ^a^	0.84 ± 0.01 ^a^

Values are expressed as mean value ± SD; *n* = 4. ^a–c^ Different letters in one row correspond to sig-nificant differences (*p* < 0.05).

## Data Availability

Data available on request.
